# The effect of fast and slow decision-making on equity–efficiency tradeoffs and moral repugnance

**DOI:** 10.1098/rsos.230558

**Published:** 2023-09-27

**Authors:** Emil Persson, Gustav Tinghög

**Affiliations:** ^1^ Division of Economics, Department of Management and Engineering, Linköping University, 58183 Linköping, Sweden; ^2^ Department of Medical and Health Sciences, The National Center for Priority Setting in Health Care, Linköping University, 58183 Linköping, Sweden

**Keywords:** intuition, deliberation, cognition, equality, efficiency, repugnance

## Abstract

Fast-and-slow models of decision-making are commonly invoked to explain economic behaviour. However, past research has focused on human cooperation and generosity and thus largely overlooked situations where there are sharp conflicts between efficiency and equality, or between efficiency and more intuitive moral values (repugnance). Here, we contribute to fill this gap in the literature. We conducted a preregistered experiment (*n* = 1500 recruited from Prolific) to assess the effects of fast, intuitive decisions, under time pressure versus slow, deliberate decisions, under time delay, on (i) people's distributional preferences and (ii) their attitudes toward repugnant transactions. The results show increased preference for equality and decreased preference for efficiency under time pressure, but no effects on moral repugnance. Exploratory analyses revealed that most of the observed treatment effects in our data were accounted for by women. Our results provide some support for theories that associate controlled cognition with concern for efficiency, and intuitive, emotional responses with inequality aversion.

## 1. Introduction

There are fundamental conflicts of value in society between material efficiency, on the one hand, and other normative claims, such as equity, need or human dignity. A distinction can be made between tradeoffs that concern only secular values, a classical example being efficiency–equality tradeoffs in the distribution of material resources; and tradeoffs that also involve more profound moral values, such as health or human life [1]. An example of the latter is whether to allow the purchase and sale of human organs to increase the stock available for transplantation, which is often considered morally repugnant. People's views of these issues, and their social preferences and moral attitudes at large, have implications for policy in important areas, such as government-funded healthcare, social welfare and taxation. An active research field has tried to understand where these preferences come from, by focusing on how cognitive processes linked to intuition and deliberation shape social decisions [2–7]. However, the main focus in this literature has been on generosity and cooperation (whether fairness is intuitive), and the cognitive foundations of the fundamental conflicts between equality and efficiency, and between efficiency and moral sacred values, have largely been neglected. In this paper, we contribute to fill this gap in the literature by investigating the role of intuition and deliberation in (i) monetary allocation decisions where there are conflicts between different social motives, primarily efficiency and equality and in (ii) attitudes toward transactions where there are conflicts between material efficiency and moral sacred values.

In the experiment, we used time pressure to invoke fast, relatively more intuitive decisions, and time delay to invoke slow, relatively more deliberative decisions. Subjects first made a series of incentivized binary allocation decisions and then responded with stated attitudes and made moral judgements (in hypothetical scenarios) about transactions that are often considered repugnant. Repugnant transactions are market exchanges that uninvolved persons want to prevent, often out of moral concerns and even though the transactions bear no negative consequences on these persons themselves [8,9]. A typical example is the buying and selling of organs for transplantation [8,10,11]. Repugnance can and often does act as a real constraint on markets [8]. We reasoned that moral repugnance and distributional preferences about monetary allocations have common features, in both a practical and theoretical sense; and that studying them jointly could generate new insights. From a practical point of view, both moral repugnance and distributional preferences concern attitudes toward outcomes where material efficiency stands in conflict with other normative values when engaging in tradeoff thinking. From a theoretical point of view, behaviour in both domains shares features that are commonly viewed as amenable to manipulations of response times, especially the reliance on negative emotion and ‘gut feelings’.

The previous literature on the cognitive basis of equity–efficiency tradeoff is limited [7]. Our current knowledge stems primarily from Capraro *et al*. [12], who studied what social motives became more prevalent in allocation decisions made under time pressure. They found that time pressure promoted egalitarianism and time delay promoted efficiency. Thus, heightened reliance on intuition seemed to make people more accepting of sacrificing efficiency for equity in distribution decisions. A similar tendency can be glanced from a few other studies that used a similar choice paradigm. Chen & Fischbacher [13] also used time pressure to investigate the cognitive processes underlying distributional preferences. However, they assessed selfish versus social motives jointly and did not test whether intuition or deliberation appeared to be selectively linked to specific social motives, such equality, efficiency or spitefulness. Krawczyk & Sylwestrzak [14] and Kessler *et al*. [15] used a two-response paradigm, where subjects submit an initial, fast, response that they can later change, if they wish. This type of design essentially tests the corrective assumption of dual-process theory [5], where deliberation overrides the intuitive response. Krawczyk & Sylwestrzak [14] found that subjects' intuitive responses tended to be less accepting of disadvantageous inequality and Kessler *et al*. [15] found that deliberation made subjects more generous when generosity was sufficiently efficient. Both of these studies are thus conceptually in line with the results in Capraro *et al*. [12].

In addition, some studies have investigated the cognitive processes underlying distribution decisions under impartiality, i.e. when making choices for other people. Schneider & Leland [16] found suggestive evidence of trait-intuition in the same direction as Capraro *et al*. [12], who also consider intuitive trait, using the cognitive reflection test (CRT), with greater emphasis on equality for low-CRT and greater emphasis on efficiency for high-CRT (see also [17]). Ueshima & Kameda [18] used mouse-tracking and found suggestive evidence that maximin concerns manifest cognitively much faster and more robustly than egalitarian or efficiency concerns, which indicates that this motive acts as a cognitive anchor in intuitive, fast responses. Hsu *et al*. [19] found neurocognitive evidence broadly consistent with the idea that equality concerns are rooted in intuitive emotional processing.

Our paper builds on a large literature that investigated whether generosity and cooperation are intuitive responses for most people [2–4,7,20–28]. There is currently mixed evidence on this issue [7,29–31] and recent work has emphasized heterogeneity, pointing to the apparent coexistence of selfish and prosocial intuitions [5,13,32–34]. Similarly, for attitudes and judgements about moral repugnance, no study that we are aware of has investigated the underlying role of intuition and deliberation; but there is a large tangential literature on judgements in stylized sacrificial dilemmas that typically centre on the question whether to kill one person to save a greater number of persons. In that context, there is converging evidence that intuition favours deontological judgements (e.g. harmful actions are always wrong no matter the consequences) in moral dilemmas involving direct harm, but not in those involving harm as a side effect [7].

## 2. Theory and hypotheses

Intuition and deliberation are often conceptualized as *dual* cognitive processes, commonly described as two types, or two systems, with decision-making viewed as the resulting outcome from the interaction of these two systems. Intuitive processes are characterized as fast, automatic, effortless and emotional. By contrast, deliberative processes are slower and more controlled, effortful and reflective; they load on working memory and support hypothetical thinking and mental simulation [6,35–41]. In the experiment, we manipulate response times to invoke relatively more intuitive decisions, using time pressure, and relatively more deliberative decisions, using time delay. On this basis, we developed hypotheses about choice patterns that would be expected when either intuitive or deliberative processes can be assumed to have relatively more influence over decisions.

Intuitive processing generates default responses, which can be described as intuitive thoughts that come to mind spontaneously. They are routinely produced, without effort or intent. The mental content of these thoughts is based on attributes that are easily and automatically perceived, such as size, distance, mood, affective valence and the evaluation of objects as good or bad. Since these attributes are highly accessible to decision-makers, they play an important role in judgement and choice based on intuitive processing [38,42].

Two prominent attributes that are intuitively accessible at the point of decision-making and thus thought to be influential in choice based on intuition are (i) change or relative difference, e.g. between two possible payoffs, and (ii) affective valence, e.g. psychological pain of receiving less than someone else. This ties intuitive decision-making to theories of outcome-based social preferences emphasizing people's dislike of disadvantageous inequality [43,44], via both negative affect and perceptual salience of relative payoffs. Negative affect toward those who have more has been suggested as a strong reinforcer of inequality aversion [45,46], as opposed to fairness concerns related to advantageous inequality, which are thought to be more rooted in higher-level cognitive processes capable of mental simulation [46]. In addition, by the notion of perceptual salience, an allocation where the decision-maker receives $10 and another person receives $18 is arguably more salient than an allocation where the two individuals receive $10 each (using e.g. the conceptualization of salience in Bordalo *et al*. [47]); and if something bad (for me) is salient it likely triggers avoidance. Taken together, we, therefore, expect increased aversion toward disadvantageous inequality under intuition, and thus increased preference for egalitarian allocations over more efficient but unequal allocations.

Hypothesis 1: Choice patterns consistent with egalitarianism are more common under time pressure than time delay.

The flipside of our reasoning behind Hypothesis 1 (egalitarianism is intuitive) is that efficiency concerns will be given more weight in participants' decision-making when given enough time to deliberate, because it is less salient and not as emotionally loaded. In addition, some cognitive effort is arguably required to first compute the sums of two different allocations (to assess efficiency) and then evaluate, and possibly override, the initial gut reaction of equalizing payoffs due to intuitive dislike for disadvantageous inequality. This line of reasoning leads to our second hypothesis.

Hypothesis 2: Choice patterns consistent with efficiency concerns are more common under time delay than time pressure.

Moral repugnance is closely linked to negative emotion and moral outrage [9]. We thus expect increased aversion toward repugnant behaviours under intuition, where affective gut reactions are thought to play an important role; and, vice versa, greater acceptance following deliberation, when people have had more time to assess the tradeoffs and potential benefits for the people directly involved in the transactions. This line of reasoning builds on Greene and colleagues’ dual-process theory of emotional engagement in moral judgement, where automatic emotional processing is a central component of deontological judgement, and effortful and controlled deliberative processing is more involved in consequential decision-making and utilitarian judgement [48,49]. In the moral repugnance part of our experiment, subjects made a series of judgements in hypothetical scenarios and responded to attitude questions in situations that involved a tradeoff between efficiency (in general terms) and moral, sacred values; for example, whether to allow buying and selling of kidneys for transplantation. Our third hypothesis is the following:

Hypothesis 3: There is increased aversion toward potentially efficient but morally repugnant transactions under time pressure compared to time delay.

## 3. Material and methods

The study was preregistered at https://osf.io/snauv/. Data, analysis codes and experimental materials are available via the project's OSF repository (link above) [50]. All conditions run and measures collected are reported; see electronic supplementary material for complete transcript of instructions and decision screens. Sample size was determined in advance; we reasoned that *n* = 1500 would give us sufficient power to detect reasonably small effects, e.g. greater than 80% power for Cohen's *h* = 0.15 (at *α* = 0.05), which is roughly half the size of the main effect found in Capraro *et al*. [12] (based on our own calculations for observed differences in egalitarian and efficiency profiles).

### 3.1. Preliminary study using cognitive load

In addition to the study reported in this paper, we had previously conducted a preliminary experiment using similar materials but with cognitive load (versus control) instead of time pressure (versus time delay). We had planned to report these two studies jointly. However, the quality of data from that study was not as good as we hoped and we are, therefore, reluctant to include it here. To maintain transparency we have made the data, materials and results from that experiment available via this project's OSF repository (link above).

In short, our main issue with the data is that an unusually large number of participants failed the attention check, even in the control condition (the attention check appeared while under load), also considering that the experiment was conducted in person, not online; 22% failed in the control condition and 41% in the cognitive load condition. In addition, a slightly larger (we would have expected smaller) proportion of participants chose inconsistently in the control condition compared to cognitive load, 18% versus 15%. Together these numbers indicate that something with this data collection was not working as intended.

Even so, we carried out all the planned main analyses and there were no statistically significant differences between cognitive load and control. For reasons just described we are reluctant to interpret or discuss these results further; taken at face value, a cautious interpretation would be that they indicate an absence of large effects of cognitive load on distributional preferences and moral repugnance.

### 3.2. Participants

One thousand five hundred and one English-speaking volunteers (56% female, mean (s.d.) age 34.5 (12.6) years) were recruited from Prolific [51]. Participants were randomized to a *time pressure* condition (*n* = 751) or a *time delay* condition (*n* = 750) using a between-subjects design. The groups were similar in terms of age and sex. Most participants passed the attention check and responded inside the time limit on all decisions or on all but one decision (table 1). All participants gave informed consent prior to participation.

**Table 1.** Sample characteristics.

	time pressure *n* = 751	time delay *n* = 750
female	55%	57%
age, mean (s.d.), range	34.5 (12.3), 18–79	34.5 (13.0), 18–76
clear attention check	94%	96%
subject respects time limit (14 decisions in total)		
never misses time limit	78%	100% by definition
misses time limit once	18%	—
misses time limit two or more times	4%	—

### 3.3. Experimental design and procedure

Participants were randomized to a *time pressure* condition or a *time delay* condition using a between-subjects design and they stayed in the assigned condition throughout the experiment (thus participants either made all choices for all tasks under time pressure, or made all choices for all tasks under time delay). In the time pressure condition, subjects were instructed to ‘try to make each choice as fast as possible’. There was a set time limit for each decision and a timer on the screen indicated how much time they had left. We used a time limit of 7 s for binary distribution decisions, 10 s for one-sentence moral statements where subjects indicated level of agreement or disagreement and 35 s for hypothetical scenarios (moral dilemmas) that typically comprised five to seven sentences. If no choice had been made before the time was up, subjects were automatically prompted with a reminder to ‘please respect the time limit on the remaining questions’. They could then submit their response and move on to the next item. In the time delay condition, subjects were instead instructed to ‘think carefully through each decision problem’ and they could not submit their choice and move on to the next item before a set time had passed. The set times were the same as the time limits in the time pressure condition (7, 10 and 35 s).

The purpose of the experimental manipulation was to invoke relatively more intuitive decisions with time pressure and relatively more deliberative decisions with time delay. Deciding on time limits, our goal was to strike a good balance between stringency and error control. The time limit should be salient, and stringent enough to speed up decision times considerably, but without introducing too much noise in decision-making. The chosen time limits, 7 s for binary decisions and 35 s for dilemma-type questions, have worked well for us in the past [4,52–54].

There were three parts of the experiment, described below in detail. First, subjects made six incentivized binary distribution decisions, then they indicated agreement or disagreement with four moral statements (three of these were short and thus on a 10 s time limit, and the fourth was long and thus on a 35 s limit), and finally they responded to four hypothetical moral scenarios (35 s time limit). As described above, participants were randomized to a time pressure condition or a time delay condition and stayed in the assigned condition throughout the experiment. Thus, the time limits mentioned here were an upper limit for participants in the time pressure condition and a lower limit (amount of delay) for participants in the time delay condition. At the end, subjects responded to an attention check, background questions, and some additional measures collected for exploratory purposes. The experiment lasted approximately 10–15 min and participants were paid on average £2.30 (mean hourly rate of approx. £12). A complete transcript of experimental instructions and decision screens can be found in the electronic supplementary material.

### 3.4. Distributional preferences

In this part, participants made six binary decisions (in random order) on how to allocate a sum of money between themselves and another anonymous participant they were randomly matched with. After the study, one decision (for each matched pair of participants) was randomly selected for real payment. The six decision problems were the same as in Capraro *et al*. [12], shown in table 2. All decision problems had an egalitarian option with equal payoffs and another option with some level of inequality, either advantageous or disadvantageous from the perspective of the decision maker. The option with disadvantageous inequality was always more efficient (problems three, four and six). The option with advantageous inequality was either less efficient than the egalitarian option, in problems one and five, or equally efficient, in problem two, which is also a binary dictator game (zero-sum; e.g. [55]).

**Table 2.** Decision problems used in the experiment to elicit distributional preferences.^a^

	option A (DM, other)	option B (DM, other)	motives consistent with option A	motives consistent with option B
1	10, 10	10, 6	efficiency, egalitarian	spiteful
2	10, 10	16, 4	efficiency, egalitarian	efficiency, spiteful, self-interest
3	10, 10	10, 18	egalitarian, spiteful,	efficiency
4	10, 10	11, 19	egalitarian, spiteful	efficiency, self-interest
5	10, 10	12, 4	efficiency, egalitarian	spiteful, self-interest
6	10, 10	8, 16	egalitarian, self-interest, spiteful	efficiency

^a^Based on table 1 in Capraro *et al*. [12]. DM indicates decision maker.

We followed Capraro *et al*. [12] and labelled individual choices along four different preference categories: *efficiency* for choices that maximize the total joint payoff, *egalitarian* if they minimize payoff inequality, *spiteful* if they maximize their own relative payoff by minimizing the other's payoff and *self-interested* if they maximize their own payoff. We used two different methods to classify each subject's overall choice pattern (i.e. the profile from their specific choices in the six decision problems) into one or more of these four categories: (i) a model-based classification using a generalized Fehr–Schmidt model [44] and (ii) a choice-based classification using a standard approach from the literature on social value orientation [12,56].

The model-based method classifies choice profiles to preference categories based on consistency with specific parametrizations of the generalized Fehr–Schmidt utility function, $U_{i}(x_{i},x_{j})=x_{i}-\alpha_{i}$ $\max\{x_{j}-x_{i},0\}-\beta_{i}\max\{x_{i}-x_{j},0\}$ for individuals *i*,*j*, where the parameters $\alpha_{i}$ and $\beta_{i}$ capture *i*'s aversion to disadvantageous and advantageous inequality, respectively. For example, the *egalitarian* preference category requires minimization of payoff inequality and thus the accompanying parametrization is $\alpha_{i}\geq 0$ and $\beta_{i}\geq 0$, with at least one strict inequality; any choice profile that is consistent with this parametrization (e.g. ABAABA, where option A was chosen on the first decision problem, B on the second, and so on) is thus classified as egalitarian. And similarly for the other three preference categories. By contrast, the choice-based method classifies choice profiles in accordance with a specific preference category if at least two-thirds of their choices are consistent with that motive, as defined in table 2. For example, choice profile BBAAAB is in line with spiteful using this classification, because the options chosen in the first four decision problems are in line with this motive, even though the fifth and sixth decisions are not.

Each approach gives four main dependent variables, one for each preference category (*efficiency*, *egalitarian*, *spiteful* and *self-interest*). These variables are coded = 1 if subjects' choices are consistent with that category and = 0 otherwise. The choice-based method works for all possible profiles, i.e. for any combination of options chosen in the six decision problems, and each dependent variable can thus be consistently coded as = 1 or = 0; but the model-based method has a consistency requirement in that subjects’ choices must be consistent with *some* parametrization of the generalized Fehr–Schmidt utility function, otherwise the observation is dropped from analysis. See electronic supplementary material for more details about the classification.

### 3.5. Moral repugnance

This part of the experiment was designed to elicit participants' views about behaviours that are often considered morally repugnant. Subjects made a series of judgements in hypothetical scenarios and responded to attitude questions in situations that involved a tradeoff between efficiency (in general terms) and moral, sacred values.

For example, in one of the scenarios, subjects were asked to make a judgement about the prospect of selling kidneys. The scenario read: *As diabetes becomes more common, more kidneys will fail. The alternatives are: death, dialysis, kidney transplant. Dialysis is very expensive. Only the very richest countries can dialyze all who need it. Moreover, dialysis is very burdensome for the patient and decrease quality of life significantly. Each year many people die waiting for a kidney transplant. If kidney sales were permitted, there would be no shortage of kidneys and the number of people needing dialysis would drop sharply. However, mostly the poor would sell their kidneys. Should we allow that people sell and buy kidneys?* There were three other similar scenarios (see electronic supplementary material for details) where subjects made binary moral judgements; one response alternative was always more efficient but less respecting of moral sacred values. The primary dependent variable (*moral judgement*) is the proportion of efficiency-oriented choices made by each participant.

Subjects also indicated their level of agreement with four (additional) statements that involved a tradeoff between efficiency and moral sacred values on a 7-point Likert scale [57]. For example, one of the statements read: *Some aspects of humanity are sacred and should not be violated no matter the possible material gain*. There were three other similar statements (two of them were short and one was longer); see electronic supplementary material for details. The primary dependent variable (*moral values*) is the average rating for each participant.

### 3.6. Statistical analysis

The main analysis plan was specified in advance; see the preregistration for details. In our main confirmatory analyses, we tested for a difference in proportion across conditions for each of the four dependent variables representing distributional preference categories (*efficiency*, *egalitarian*, *spiteful*, *self-interest*) using two-proportion *z*-tests as well as linear regressions with age and sex as additional covariates. We followed our preset contingency plan and assessed both the model-based and the choice-based classifications, given that the rate of model-inconsistent choosing was 16% and thus fell inside the 15–25% interval. We followed up with planned secondary analyses that assessed the robustness of our main findings. We also conducted planned secondary analyses of decisions in the second decision problem (which is a binary dictator game) and an unplanned exploratory analysis that displayed differences in choice rates for each of the six distribution decision problems. For moral repugnance, our main confirmatory analyses entailed testing for differences in (i) the average proportion of efficiency-oriented choices (moral judgement) and (ii) the average efficiency-oriented ratings (moral values) across conditions, using independent samples *t*-tests as well as linear regressions with age and sex as additional covariates. We followed up with planned robustness checks and secondary analyses that compared mean responses in each of the eight scenarios and statements. Finally, for both distributional preferences and moral repugnance we conducted exploratory analyses of potential differences between men and women in response to treatment (these final analyses were contingent on observed data and not hypothesis driven, and not preregistered) and we assessed robustness when removing the fastest responders in the time pressure condition.

## 4. Results

### 4.1. Distributional preferences

Eighty nine per cent of participants in the time pressure condition responded before the time limit (seven seconds) on all six distribution decisions, and an additional 10% missed the time limit only once. The mean (s.d.) response time was 3.9 (1.0) s in time pressure, excluding choices that were outside the time limit, approximately 2%; and 10.4 (6.2) s in time delay. See electronic supplementary material, figure S1, for the distribution of response times. The rate of model-inconsistent choosing was 16% in total; 18% in time pressure and 13% in time delay (proportions test, diff = 0.05, s.e. = 0.02, *z* = 2.40, *p* = 0.02).

**Figure 1.**
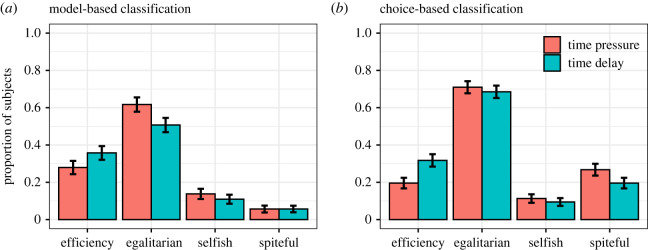
Type classification based on distributional choices. For the model-based classification, inconsistent subjects are excluded, thus *n* = 616 in time pressure and *n* = 649 in time delay. For the choice-based classification, all subjects are included (*n* = 751 in time pressure and *n* = 750 in time delay). Error bars indicate 95% confidence intervals from two-sample proportion tests.

The main results from our confirmatory analyses can be seen in figure 1. The share of subjects classified as *efficiency* oriented was 8–12% points smaller under time pressure compared to time delay (model-based, diff = −0.08, s.e. = 0.03, *z* = −2.98, *p* = 0.003, 95% CI, −0.13, −0.03; choice-based, diff = −0.12, s.e. = 0.02, *z* = −5.39, *p* < 0.001, 95% CI, −0.17, −0.08). By contrast, a larger share of subjects were classified as *egalitarian* under time pressure compared to time delay using the model-based method (diff = 0.11, s.e. = 0.03, *z* = 3.94, *p* < 0.001, 95% CI, 0.06, 0.16); but the difference was smaller and insignificant using the choice-based method (diff = 0.02, s.e. = 0.02, *z* = 1.03, *p* = 0.30, 95% CI, −0.02, 0.07). Few subjects were classified as *self-interested* and the estimated differences between conditions were small and insignificant (model-based, diff = 0.03, s.e. = 0.02, *z* = 1.55, *p* = 0.12, 95% CI, −0.01, 0.06; choice-based, diff = −0.02, s.e. = 0.02, *z* = 1.18, *p* = 0.24, 95% CI, −0.01, 0.05). Finally, a larger share of subjects were classified as *spiteful* under time pressure compared to time delay using the choice-based method but not with the model-based method (model-based, diff = −0.00, s.e. = 0.01, *z* = −0.01, *p* = 0.99, 95% CI, −0.03, 0.03; choice-based, diff = 0.07, s.e. = 0.02, *z* = 3.29, *p* = 0.001, 95% CI, 0.03, 0.11). The results were similar when we used linear regression and included age and sex as additional covariates and these models were robust to excluding subjects who were inattentive, did not respond on time, or failed the model-based consistency requirement (see electronic supplementary material, tables S1–S4, for details). The results were also robust to excluding the fastest responders in the time pressure condition (see electronic supplementary material, table S6, for details).

Planned regressions (mentioned above and reported in electronic supplementary material, table S1) revealed a substantial difference between men and women, where women were less likely than men to be classified as efficiency oriented, and more likely to be classified as egalitarian. We followed up with exploratory analyses to assess potential differences between men and women in response to treatment, i.e. time pressure versus time delay. Using the model-based approach, we found that time pressure made men 1.4% points less likely to be classified as efficiency oriented, compared to men in the time delay condition; for women, the corresponding effect was larger and amounted to a 12.6% point difference between time pressure and time delay (diff women versus men = 0.112, s.e. = 0.052, *t*_1260_ = 2.15, *p* = 0.031, 95% CI, 0.214, 0.010; see electronic supplementary material, table S5, for details). Similarly, men were 3.3% points more likely to be classified as egalitarian under time pressure compared to time delay; for women the corresponding effect was 16.8% points (diff women versus men = 0.135, s.e. = 0.054, *t*_1260_ = 2.49, *p* = 0.013, 95% CI, 0.029, 0.241). Thus, using the model-based method, most of the observed treatment effect seems to be accounted for by women. For the choice-based method, the pattern was qualitatively similar, e.g. point estimates for the effect of treatment were larger for women, but less pronounced, and the estimated interaction effects were not statistically significant.

We conducted exploratory analyses of the difference across conditions for each of the six underlying distribution decisions (games). The results are shown in table 3. For all three games where one of the options results in disadvantageous inequality for the decision maker (i.e. he or she gets a lower payoff than the matched participant; games three, four and six), time pressure reduced the likelihood that this option was chosen, despite the fact that this is the more efficient option in all three cases. This finding supports the interpretation that time pressure increased people's propensity to sacrifice efficiency for equity, even at substantial costs for other people; e.g. the payoff to the other participant is cut by almost 50% in games three and four.

**Table 3.** Distributional choices for each decision problem, by condition.^a^

(DM, other)	time pressure *n* = 751	time delay *n* = 750	*z*-test difference
10, 10 versus 10, 6	0.88	0.89	*p* = 0.42
10, 10 versus 16, 4 (dictator game)	0.64	0.65	*p* = 0.68
10, 10 versus 10, 18	0.64	0.56	*p* = 0.002
10, 10 versus 11, 19	0.62	0.52	*p* < 0.001
10, 10 versus 12, 4	0.70	0.77	*p* = 0.002
10, 10 versus 8, 16	0.92	0.86	*p* < 0.001

^a^For each decision problem (game), the table shows the proportion of subjects who chose the alternative with equal payoffs (10, 10) in each condition. DM indicates decision maker.

In the table, we can also see that very few subjects chose the spiteful option B (10, 6) in the first game. This explains why so few subjects were classified as spiteful using the model-based method, because, with that method, choosing option B in the first game is a necessary condition for being classified as spiteful. By contrast, the choice-based method tolerates some degree of inconsistency, and thus more subjects are classified as spiteful using that method. Part of the effect of time pressure on egalitarian preference seen with the model-based method is thus likely captured by spitefulness with the choice-based method. Indeed, of the 348 subjects classified as spiteful using the choice-based method, 47% were classified as egalitarian using the model-based method.

In our preregistration, we had specified follow-up analyses for the second game, which is a binary dictator game. As already reported in table 3, there was no discernible difference in choice behaviour between time pressure and time delay for this game. A linear regression that included age and sex as covariates yielded similar results (*b* = 0.008, s.e. = 0.024, *t*_1497_ = 0.34, *p* = 0.73, 95% CI, −0.039, 0.056), and the estimated interaction effect between sex and condition was small and insignificant (*b* = −0.009, s.e. = 0.050, *t*_1496_ = −0.19, *p* = 0.85, 95% CI, −0.107, 0.088). Thus, we found no effect of time pressure on decisions in the dictator game.

### 4.2. Moral repugnance

Eighty seven per cent of participants in the time pressure condition responded before the time limit on all eight questions (statements and scenarios), and an additional 11% missed the time limit only once. On questions with a 10 s time limit, the mean (s.d.) response time was 7.1 (1.6) s in time pressure, excluding choices that were outside the time limit, approximately 3%; and 17.3 (31.6) s in time delay. On questions with a 35 s time limit, the mean (s.d.) response time was 21.1 (5.3) s in time pressure, excluding choices that were outside the time limit, approximately 1%; and 49.0 (32.2) s in time delay. See electronic supplementary material, figure S2, for the distribution of response times.

**Figure 2.**
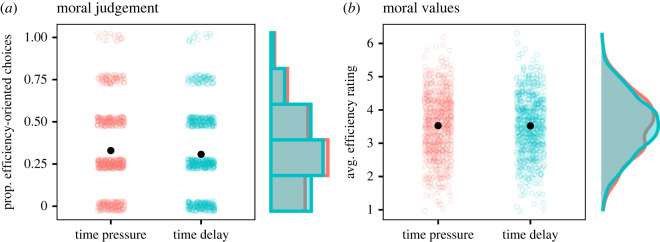
Moral judgement and moral values, by condition. Moral judgement (*a*) is the proportion of efficiency-oriented choices made by individuals across four binary-response scenarios. Stated moral value (*b*) is the average efficiency rating for each individual from four scenarios, each rated on a 1–7 scale. Black dots indicate sample means, 95% confidence intervals are (0.31, 0.35) and (0.29, 0.33) in time pressure and time delay for moral judgement, and (3.46, 3.59) and (3.46, 3.58), respectively, for moral values. Sample size is *n* = 751 in time pressure and *n* = 750 in time delay.

The main results from our confirmatory analyses can be seen in figure 2. The estimated mean difference between time pressure and time delay was small and insignificant, both for moral judgements in hypothetical scenarios and for stated moral values (moral judgements, *b* = 0.022, s.e. = 0.013, *t*_1499_ = 1.70, *p* = 0.09, 95% CI, −0.003, 0.048; moral values, *b* = 0.007, s.e. = 0.046, *t*_1499_ = 0.15, *p* = 0.88, 95% CI, −0.084, 0.098). The results were similar when we used linear regression and included age and sex as additional covariates and these models were robust to excluding subjects who were inattentive or did not respond on time (see electronic supplementary material, tables S7 and S8, for details). The results were also robust to excluding the fastest responders in the time pressure condition (see electronic supplementary material, table S10, for details). Taken together, we did not find increased aversion toward potentially efficient but morally repugnant transactions under time pressure compared to time delay.

Planned regressions (mentioned above and reported in electronic supplementary material, table S7) revealed a substantial difference between men and women, where women were less likely to accept morally repugnant actions, in both the scenarios (moral judgement) and statements (moral values). We followed up with exploratory analyses to assess potential differences between men and women in response to treatment, i.e. time pressure versus time delay. For moral judgements, there was no difference between men and women in response to treatment; the estimated interaction between condition and sex was small and insignificant (*b* = −0.030, s.e. = 0.025, *t*_1496_ = −1.19, *p* = 0.23, 95% CI, −0.080, 0.020; see electronic supplementary material, table S9, for details). But for moral values, we found a significant interaction effect, with effects of time pressure versus time delay going in different directions for men and women (*b* interaction = −0.272, s.e. = 0.089, *t*_1496_ = −3.05, *p* = 0.002, 95% CI, −0.446, −0.097). Men on average increased their point score with 0.15 units (the outcome is measured on a 1–7 scale), meaning that they became more accepting of morally repugnant actions under time pressure; women, in contrast, on average decreased their point score with 0.12, meaning that they became less accepting of morally repugnant actions. Whereas the magnitude of each effect is small, the magnitude of the difference is more substantial, around one-fourth of the joint standard deviation (approx. Cohen's *d* = 0.3).

We also conducted exploratory analyses of the difference across conditions for each of the eight underlying questions (tables 4 and 5). The results showed a stable pattern of decision-making across the different scenarios and statements. We saw in figure 2 that the modal response for moral judgement was to endorse the morally repugnant but more efficient option (e.g. allow market for kidneys) on one out of four occasions. The disaggregated results in table 4 reveal that it was not one scenario in particular where subjects endorsed the repugnant action, but rather this seemed to vary across subjects, as indicated by the stable base rates across scenarios. For moral values, mean responses were tilted toward disagreement with morally repugnant actions on two statements, and toward slight agreement on the remaining two statements (table 5).

**Table 4.** Moral judgements in each scenario, by condition.^a^

	time pressure *n* = 751	time delay *n* = 750	*z*-test difference
liver transplant to rich man	0.27	0.26	*p* = 0.42
vaccine priority to company executive	0.27	0.21	*p* = 0.01
allow market for kidneys	0.32	0.29	*p* = 0.27
allow dwarf-tossing events	0.46	0.47	*p* = 0.66

^a^For each scenario, the table shows the proportion of subjects in each condition who chose the morally repugnant but more efficient alternative.

**Table 5.** Moral values for each statement, by condition.^a^

	time pressure *n* = 751	time delay *n* = 750	KS-test difference
material gains over sacred values	2.59 (1.37)	2.38 (1.28)	*p* = 0.09
pain acceptable for national security	3.11 (1.66)	3.02 (1.66)	*p* = 0.48
freedom to do immoral things	4.37 (1.67)	4.65 (1.56)	*p* = 0.02
vaccine utilitarian sacrifice	4.05 (1.85)	4.04 (1.86)	*p* = 1.00

^a^For each statement, the table shows the mean (s.d.) rating in each condition. Range 1–7, higher values indicate stronger agreement with efficient but morally repugnant actions. KS indicates Kolmogorov–Smirnov test for equality of distributions.

## 5. Discussion

We conducted a preregistered experiment (*n* = 1500 participants recruited from Prolific) to assess the effect of fast and slow decision-making on distributional preferences and moral repugnance. Our results are clear. We found increased preference for egalitarian outcomes and decreased preference for efficient outcomes when decisions were fast, made under time pressure, compared to decisions that were slower, made under time delay. These findings are similar to those of Capraro *et al*. [12], which suggest converging evidence for a robust influence of time pressure on monetary allocation decisions when there are conflicts between equality and efficiency. Exploratory analyses (not hypothesis driven) revealed that most of the observed treatment effects in our data were accounted for by women. There were no effects of time pressure on decisions in the binary dictator game, which is in line with recent meta-analytic evidence [23,29]. Moreover, contrary to our hypothesis for moral repugnance, there were no effects of time pressure on people's views about transactions that are potentially efficient but often considered morally repugnant. Our study contributes to the growing literature on the cognitive foundations of social preferences.

From a theoretical point of view, our findings provide some support for dual-process theories of social cognition and decision-making. Our data are consistent with the view that perception, salience and emotion are integral features of intuitive choice [38,39,42]. Of note, our results could, in principle, also be rationalized by sequential sampling theories [34,58]. For example, one could assume that people put higher weight on equality and lower weight on efficiency, meaning that time pressure would strengthen the evidence accumulated for equality and weaken the evidence accumulated for efficiency, and time delay would have opposite effects. Separating these two partly competing theoretical accounts is not straightforward, and it is beyond the scope of our paper. The fact that most of the treatment effects that we saw were accounted for by women is interesting, and one could speculate about the underlying causes. One possible interpretation goes via the emerging literature on experience effects and their impact on economic decision-making [59,60]. Based on that literature, a plausible conjecture is that women's greater exposure to inequality and discrimination in society influences how they perceive the stylized tradeoff between equality and efficiency in our experiment, and that this drives their choices when prompted to decide intuitively, under time pressure. Another potential explanation is that men and women tend (or tended) to have different roles in society and that as a consequence women more than men have internalized inequality aversion as their intuitive response [23]. On a general level, our findings are in line with previous work showing that men tend to be more efficiency oriented and women more egalitarian [61,62], and that this effect can be partly explained by gender differences in cognitive style, measured with the CRT [62].

Our failure to find the effects of time pressure on moral repugnance is somewhat surprising. Seen from the perspective of the dual-process theory of moral judgements [48,49,63], where controlled cognition is associated with utilitarian judgements and intuitive, emotional, responses are associated with deontological judgements, and given the results in the literature on sacrificial moral dilemmas (trolley problems), our hypothesis had a plausible basis. Still, there are other examples of null findings in this literature [53,64] or empirical patterns that are not fully compatible with a dual-process view of moral judgement [65,66]. And alternative theories that can rationalize some of these findings exist. According to Haidt [67], and in contrast to the dual-process view of moral judgements, the defining ambition of deliberation in moral judgement is generally not to provide accurate responses but to provide a convincing rationale for why immediate gut feelings should be respected. Controlled cognition should thus not affect judgements when strong negative emotions are in play, according to this so-called social-intuitionist model. Seen jointly with the results for distributional preferences, where we do find a clear effect for tradeoffs between disadvantageous inequality and efficiency, but not for altruistic giving (in the dictator game), also keeping in mind similar findings in Capraro *et al*. [12], a cautious interpretation would be that the distinction between intuitive and deliberative cognitive processing may be useful to characterize choices under some specific circumstances, as our results demonstrate; but not as a general guiding principle for understanding economic behaviour.

Our study has several strengths, including a preregistered analysis plan and a substantially larger sample than many of the previous studies in the literature. Still, there are limitations that should be noted. Knowing how people respond to time pressure (or time delay) is useful in itself, because some economic decisions are made under such circumstances. Interpreting our results in this way is relatively unproblematic, given the apparent quality of our data, e.g. most participants make consistent choices and respect time limits. However, it is potentially more problematic to infer that treatment effects are due to specific cognitive processes, as we hypothesize, because we then make assumptions about how manipulation of response times influences cognitive states. There are open questions concerning for example how fast the different processes are and how we should match this with specific time limits used in experiments [68], and also how to separate increased errors and thus noise from directed intuition. Whereas time pressure is an established and commonly used method to invoke relatively more intuition or deliberation, in economics as well as psychology and neuroscience [7], our results should be interpreted with some caution keeping these limitations in mind.

A potential drawback of our design is that we used fixed time limits and thus very fast responders might not have felt that they were under time pressure when making their choices. A different type of design (e.g. using gradual incentives [32]) might have been more suitable in this respect. Still, a substantial proportion of responses were made with only a few seconds left of the allowed decision time, at least for the short items with 7–10 s time limit. For these responders, the approaching time limit was arguably salient when making their choices. We also note that the main results were robust to excluding the fastest responders in the time pressure condition. For the longer moral judgement questions, the distribution of response times looks a bit different (figures are in electronic supplementary material). Here the median response was 21 s and thus more than 10 s before the time limit. It is possible that the manipulation of response times was less effective in this case. For the moral repugnance part, two additional limitations can be noted. First, it is possible that some of the longer scenarios were presented in a manner that induced reflective thinking (e.g. emphasizing the high cost of dialysis in the scenario about organ sale), thus possibly weakening the effect of the time pressure manipulation in these cases. Second, the order of tasks was not randomized (although items within each task were). Again this might have contributed to a somewhat weaker manipulation for this part compared to the distributional preference part, which always came first.

From a practical point of view, our results suggest that efficiency losses can be avoided if deliberation is encouraged in situations where otherwise fast, intuition-driven decisions are the norm. One reason could be that people may view excessive pondering as a signal of calculated greed or distrust (e.g. [69,70]). In addition, external factors such as sleep deprivation, choice overload, fatigue, hunger or pain may create more challenging decision environments, and, as a consequence, increased reliance on decision-making processes that are consistent with intuition rather than deliberation [71–76]. Taken at face value, our results, therefore, also suggest that social outcomes might be affected if decisions are taken under such circumstances, and that there is scope for designing institutions to mitigate these effects.

## Data Availability

The study was preregistered at https://osf.io/snauv/; data, analysis codes and electronic supplementary material (including experimental instructions and decision screens) can be accessed via this link. The data are provided in electronic supplementary material [77].
